# Pressure-Driven Piezoelectric Sensors and Energy Harvesting in Biaxially Oriented Polyethylene Terephthalate Film

**DOI:** 10.3390/s24041275

**Published:** 2024-02-17

**Authors:** Romana Stepancikova, Robert Olejnik, Jiri Matyas, Milan Masar, Berenika Hausnerova, Petr Slobodian

**Affiliations:** 1 Centre of Polymer Systems, University Institute, Tomas Bata University, Trida T. Bati 5678, 76001 Zlin, Czech Republic; danova@utb.cz (R.S.); olejnik@utb.cz (R.O.); matyas@utb.cz (J.M.); masar@utb.cz (M.M.); hausnerova@utb.cz (B.H.); 2 Department of Production Engineering, Faculty of Technology, Tomas Bata University in Zlin, Vavreckova 5669, 76001 Zlin, Czech Republic; 3 Department of Physics and Materials Engineering, Tomas Bata University in Zlin, Vavreckova 5669, 76001 Zlin, Czech Republic

**Keywords:** flexible sensor, piezoelectricity, harvesting, biaxially oriented polyethylene terephthalate

## Abstract

This study reports the possibility of using biaxially oriented polyethylene terephthalate (BOPET) plastic packaging to convert mechanical energy into electrical energy. Electricity is generated due to the piezoelectricity of BOPET. Electricity generation depends on the mechanical deformation of the processing aids (inorganic crystals), which were found and identified by SEM and EDAX analyses as SiO_2_. BOPET, as an electron source, was assembled and tested as an energy conversion and self-powered mechanical stimuli sensor using potential applications in wearable electronics. When a pressure pulse after pendulum impact with a maximum stress of 926 kPa and an impact velocity of 2.1 m/s was applied, a voltage of 60 V was generated with short-circuit current and charge densities of 15 μAcm^−2^ and 138 nCm^−2^, respectively. Due to the orientation and stress-induced crystallization of polymer chains, BOPET films acquire very good mechanical properties, which are not lost during their primary usage as packaging materials and are beneficial for the durability of the sensors. The signals detected using BOPET sensors derived from pendulum impact and sieve analyses were also harvested for up to 80 cycles and up to 40 s with short-circuit voltages of 107 V and 95 V, respectively. In addition to their low price, the advantage of sensors made from BOPET plastic packaging waste lies in their chemical resistance and stability under exposure to oxygen, ultraviolet light, and moisture.

## 1. Introduction

Piezoelectric materials convert mechanical energy into electrical energy. Among the most promising materials, piezoelectric ceramics, polymers, and polymer composites are notable for their versatility. They possess key attributes, such as flexibility, lightweight nature, effective processability through polymer processing, adaptability in design, and recyclability. This flexibility and adaptability render polymer composites particularly well-suited for applications such as sensors and energy harvesting devices [1,2,3,4,5]. Sensors constructed from these materials exhibit sensitivity to mechanical stimuli and can be integrated into self-powered systems and smart devices for various technical applications [2], including medical diagnostics [3], structural health monitoring [4], and wearable applications [5].

The concept of piezoelectric polymer materials represents the use of piezoelectric polymers such as poly(vinylidene fluoride) (PVDF) [6,7,8] or copolymers such as poly(vinylidene fluoride-trifluoroethylene) (P(VDF-TrFE)) [9] or poly(vinylidene fluoride-co-hexafluoropropylene) (P(VDF-HFP)) [10]. In addition, the piezoelectric polymer can be mixed with a non-piezoelectric polymer, such as polyborosiloxane, to improve flexibility for detecting stimuli such as bending, shock, vibration, and breathing [11]. Another possibility is the preparation of piezoelectric composites consisting of piezoelectric ceramic particles distributed in a polymer matrix [12]. The polymer matrix can represent both piezoelectric polymers with added piezoelectric particles and common non-piezoelectric polymers with active piezoelectric particles. The first group can be represented by a PVDF matrix embedded with Cs_2_AgBiBr_6_ [13], nanostructured Zn–Fe_2_O_3_ nanoparticles [14], PVDF loaded with ZnO/ZnS core–shell nanoparticles for sustainable, wearable self-powered electrical devices [15], and BaTiO_3_ particles dispersed in a piezoelectric polymeric PVDF-TrFE matrix providing flexibility and processability [16], all working as self-powered devices. The second group includes quasi-static pressure sensors or switches made from a polyamide-6 matrix with piezoelectric lead zirconate titanate (PZT) [17], PZT-porous polyurethane (PU) composites [2], calcium-modified lead titanate ceramics, and the thermoplastic polymer polyether ketone [18]. In this respect, a promising potential lies in the plastic waste that contains piezoelectric particles as the process or functional additives, as recently reported [19].

Piezoelectric generators, known as PENG, which are employed in various sectors, serve for energy harvesting and simultaneous deformation detection. Their applications include consumer goods, medical devices, engineering, automotive, aerospace, industrial settings, and security [20,21], as well as non-traditional applications such as piezoelectric harvesting generators for oceanic vehicles [22]. Polymer-based harvesting systems can incorporate PVDF thin films [8] as piezoelectric nanogenerators with an open-circuit voltage of 0.41 V, which is approximately 12 times greater than that of neat PVDF films [14] or elastic ultrathin PVDF-TrFE films applied as wearable remote devices for monitoring human body movements [23]. Polymer composites can also be applied as flexible and easy polymer processing to fabricate BaTiO_3_ particles in PVDF-TrFE polymeric matrix composites, which provide an output voltage of 59.5 V and an output current of 6.52 μA of the PENG generator [16]. If PVDF was filled with Cs_2_AgBiBr_6_, an open-circuit voltage of 126 V, short-current density of 4.67 mA m^−2^, output power density of 0.39 W m^−2^, and the ability to light up at least 86 LED and power electronic devices such as a timer were obtained [13]. The conversion sensitivity of pure PVDF was 0.091 V/N compared to 0.153 V/N for the PVDF-ZnO/ZnS composite [15]. P(VDF-HFP) filled with polyaniline and methylammonium lead iodide (CH_3_NH_3_PbI_3_) exhibited an open-circuit piezoelectric voltage output of 5 V and an output power of 8.2 nW [10].

Biaxially oriented polyethylene terephthalate (BOPET) is produced by blown extrusion into films intended for the flexible packaging of food and technical products. Plastics are always manufactured using processing aids and additives to adjust the properties of the final product. For BOPET films, mainly SiO_2_ is used in small quantities in order to reduce friction during winding and handling. Currently, only the triboelectricity of BOPET is considered for the generation of nanogenerators [24,25]. In this study, BOPET containing SiO_2_ particles is considered as a piezoelectric composite to construct self-powered sensors of mechanical stimuli and as a source of effective electrical energy harvesting, thereby generating electrical signals.

## 2. Materials and Methods

### 2.1. Material

A Tenolan^®^ biaxially oriented polyethylene terephthalate (BOPET) film with a thickness of 50 μm was obtained from Fatra a.s., Napajedla, Czech Republic.

**Figure 1:**
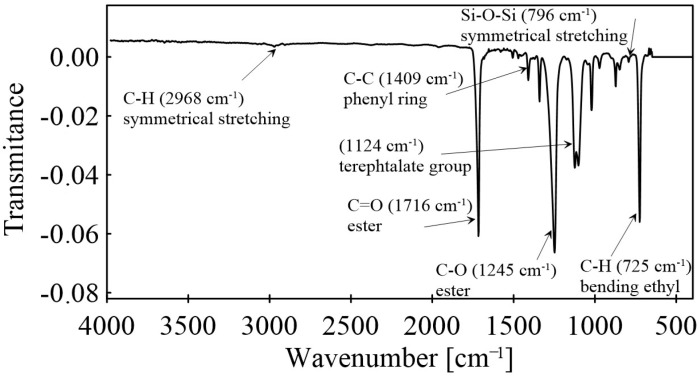
Fourier Transform Infrared (FTIR) spectroscopy spectra of BOPET film.

Fourier Transform Infrared (FTIR) spectroscopy analysis of the BOPET film was performed (FTIR Nicolet iS 5) using the Attenuated Total Reflectance (ATR) technique with a germanium crystal. The CO_2_ background spectra were subtracted and each spectrum represented an average of 64 scans. The obtained spectra (Figure 1) were compared with the HR Polymer Additives and Plasticizers library using the Omnic software (version 8), which resulted in a match of 91%.

Dielectric thermal analysis (DETA) was conducted using a broadband dielectric/impedance analyzer (Concept 50, Novocontrol Technologies GmbH & Co. KG, Montabaur, Germany). The BOPET specimen was interposed between two gold-plated electrodes with a diameter of 20 mm. The measurements of relative permittivity and dielectric loss were then carried out across a frequency range of 1 Hz to 1 MHz at room temperature.

**Figure 2:**
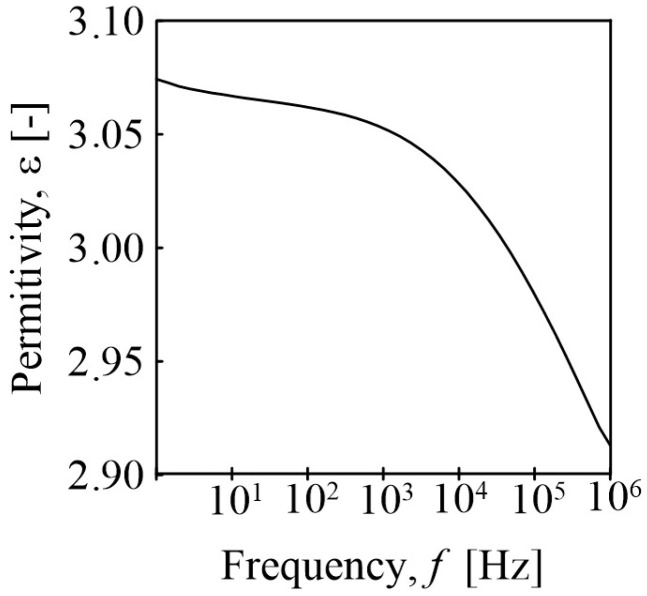
Dielectric relative permittivity of BOPET as a function of frequency at room temperature.

Dielectric thermal analysis was performed to determine the relative permittivity of the BOPET film. The results are presented in Figure 2 as frequency-dependent permittivity and dielectric loss values. The stable relative permittivity at low frequencies was 3.2. The results for our BOPET are consistent for the commercially well-known oriented polyester with the trade name Mylar^®^ with a typical BOPET film dielectric constant value of 3.2 film at 20 °C and 1 kHz [26]. Mylar, which according to a datasheet is coated with SiO_2_, was also investigated for its piezoelectric properties with a respective piezoelectric constant of 42.02 ± 10^−12^ m/V [27]. The dielectric constant of the piezoelectric layer improves the electrical output performance of piezoelectric nanogenerators [28].

The determination of the crystalline content in BOPET involved X-ray diffraction (XRD) analysis using a MiniFlexTM diffractometer (Malvern Panalytical, Malvern, UK) with CoKβ radiation at 40 kV and 15 mA. After scanning the sample in the 2θ range of 10–90°, the collected data, initially obtained using a cobalt source, were converted to a copper source using PowDLL software.

**Figure 3:**
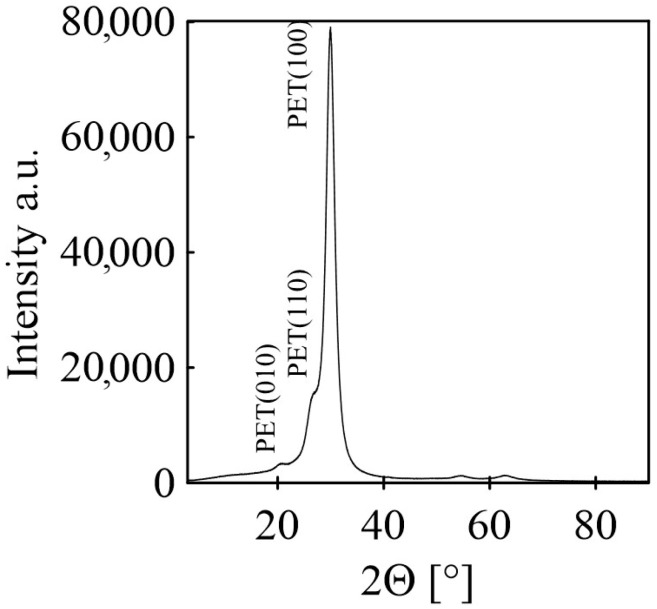
XRD analysis of the BOPET film.

The XRD analysis was aimed at validating the presence of a crystalline phase in the BOPET film, and the corresponding spectra are depicted in Figure 3. The crystallinity, denoted by *X_c_*, was calculated using the following formula [29]:

(1) $$X_{c}=\frac{\sum A_{cr}}{\sum A_{cr}+\sum A_{amr}}\times 100\;\left[\%\right]$$

where ∑*A_amr_* and ∑*A_cr_* are the total integral areas of the amorphous and crystalline diffraction peaks, respectively.

According to this analysis, the crystallinity of the BOPET was approximately 71.5%. The spectrum of the BOPET film revealed a distinctive and well-defined peak. This characteristic feature signifies meticulously organized polymer chains with a notable crystalline phase. The crystallite size calculated according to the Scherrer equation [30] for peak 100 is 34.1 Å.

**Figure 4:**
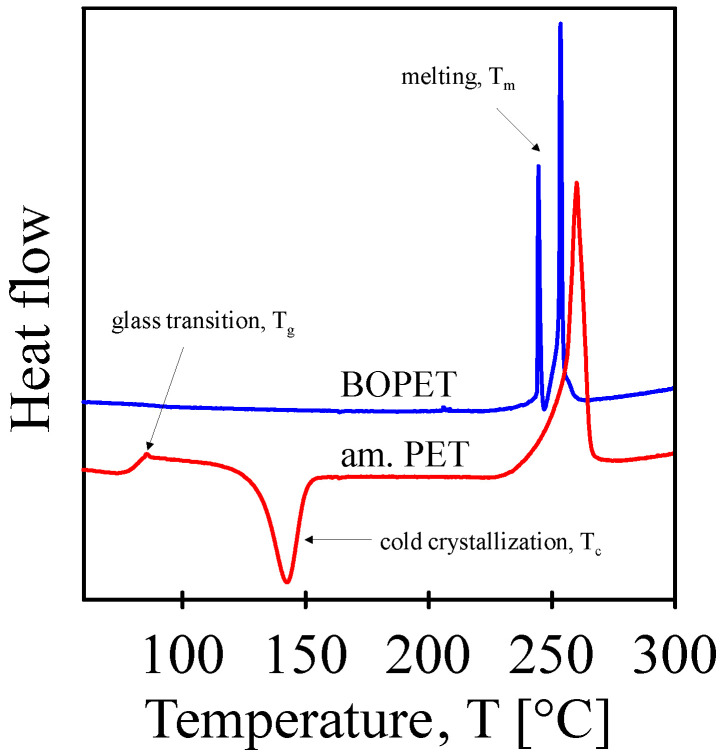
DSC analysis of BOPET film and BOPET film melted at 300 °C and then cooled by temperature jump to iced water at 0 °C.

Differential scanning calorimetry (DSC) analysis (DSC 1, Perkin Elmer, Waltham, MA, USA) was used to analyze the BOPET film and amorphous PET prepared by subjecting molten PET to a rapid temperature drop in ice water (Figure 4). The samples weighed approximately 10 mg each. The BOPET film and amorphous PET were heated from 50 to 300 °C at a heating rate of 10 °C/min.

DSC analysis confirmed that the BOPET used in this study was a semicrystalline polyethylene terephthalate polymer with parallel-oriented straight polymer chains. This was indirectly confirmed by the first measured thermo-analytical DSC curve of the BOPET film (see Figure 4), since only the melting of the crystalline phase can be observed, and no glass transition and cold crystallization were detected.

Moreover, the melting peak is bimodal, which means that there is melting of two fundamentally different types of crystals, where their structure determines the melting temperature. A thermodynamically perfect crystal has a higher melting temperature. This corresponds with the results of the XRD diffractogram, which also describes the bimodal nature of the BOPET crystalline structure. The found melting temperatures defined as the peak temperatures are 245 °C and 254 °C, with melting enthalpies 9.3 and 23.0 J/g, respectively.

The sample for the second scan prepared as amorphous PET, apart from melting, exhibited two other thermal phenomena. It is a glass transition temperature of 85 °C with a transition enthalpy of 0.4 J/g, and exothermic cold crystallization at 142 °C with −24.4 J/g. These transitions were finally followed by a simple PET melting peak at 260 °C with 44.6 J/g.

### 2.2. Characterization Methods

The examination of processing aid distribution within packing plastics involved a comprehensive analysis conducted with a scanning electron microscope (SEM), specifically the NOVA NanoSEM 450 (FEI Co., Hillsboro, OR, USA) operating at an accelerating voltage of 10 kV. Energy-dispersive X-ray spectroscopy (EDX) was employed to determine the chemical composition of the solid particles observed on the surface.

### 2.3. BOPET Self-Powered Mechanical Stimuli Sensor

**Figure 5:**
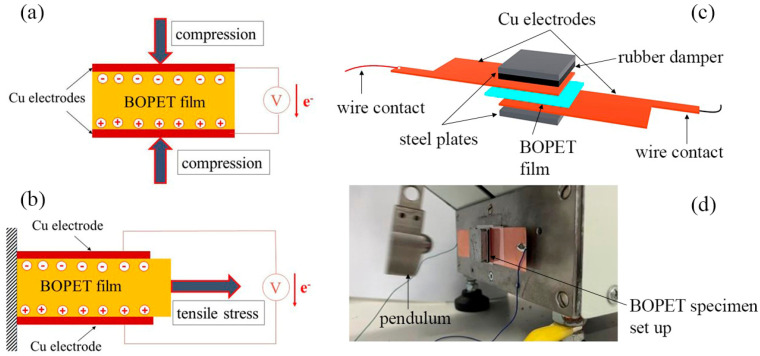
Schematic illustration of sensing systems for measuring the response of BOPET film to piezoelectric deformation by mechanical pressure stimuli (**a**) and tensile stress pulls (**b**), experimental setup for pressure mechanical loading (**c**), and arrangement of measurement (**d**).

Figure 5 illustrates the schematics and photographs of the experimental setup designed for mechanoelectrical piezo-electrification through the pressure loading of the BOPET film. Pressure pulses were administered using a rubber impact flexibility measurement apparatus (Polymer Test, Versta, Zlin, Czech Republic). In this setup, the BOPET film was positioned between two 25 mm ×25 mm copper electrodes. The applied impact energy was 0.5 J, and the impact velocity was consistently maintained at 2.1 m/s.

A strain gauge (L6D-C3-40 kg, Zemic Europe B.V., Etten-Leur, The Netherlands) integrated into the apparatus effectively gauged the force applied during pendulum impact. The strain gauge was powered by an analog converter (TZA11410, VTS Zlin s.r.o., Zlin, Czech Republic) with a ±20 mA output and 24V DC supply.

To monitor the generated piezoelectric voltage, an oscilloscope (Infinivision 1000 x-series, 4ch, 100 MHz, DSOX1204A, Keysight, Santa Rosa, CA, USA) with an input impedance of 1 MΩ was employed. To calculate the short-circuit current, Ohm’s law was applied as one cycle discharged through electrically resistive loads of 10 kΩ, measured by an oscilloscope connected in parallel to the load. Finally, the charge generated per cycle was measured using an electroscope (GRG-BTA charge sensor) connected to a LabQuest interface system (Vernier, Edu-for s.r.o., Prague, Czech Republic). The measurement for different types of mechanical loading—applied tensile stress pulls—is shown in Figure 5b.

A schematic illustration of the experimental setup for the mechanical pressure loading of the BOPET film is shown in Figure 5c. The BOPET film was sandwiched between the two copper electrodes. The electrodes were protected by metal plates (25 mm × 25 mm) against damage from pendulum strikes induced by the equipment to test the impact flexibility of the rubbers. The photograph in Figure 5d shows the arrangement of measurements. The capacitance of the sensor was approximately 18 nF.

The BOPET film was stressed under tension when the stress pulls were derived from the defined free fall of the weight. The BOPET film was placed between two copper electrodes with a width of 20 mm and a length of 40 mm, with the possibility of free movement via deformations along the electrode length. The tensile pulse in the film was derived by free-falling weights along a 20 cm track with weights of 103, 237, and 325 g, respectively. The fall velocity was 2 m/s, and the maximum tensile stresses achieved by the free fall were 30.3, 46.7, and 76.6 MPa, respectively.

Further testing of the arrangement with the potential to be a self-powered mechanical stimulus sensor was performed in the following four arrangements. The first example is the tapping of a finger onto a sensor. Second, an analytical sieve shaker (Retsch AS200; Verder Scientific, Haan, Germany) was used as the vibration source. The third example represents the response to the impact of a glass ball weighing 52 g at a speed of 4.1 m/s. Finally, the vibrational pressure stress derived from the sonotrode of the Dr. Hielscher GmbH apparatus, UP200S (200 W, 24 kHz), was tested.

### 2.4. Energy Harvesting

**Figure 6:**
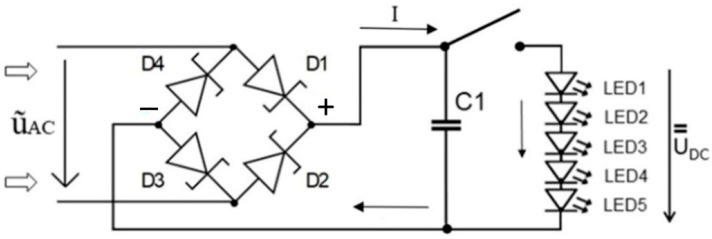
Schematic diagram of energy harvester incorporating the Graetz bridge based on Schottky diodes with storage capacitor and five LED diodes.

The Graetz bridge incorporates a quartet of Schottky diodes with a diode opening voltage of 1.5 V, as shown in Figure 6 (according to [19]). These diodes played a pivotal role in transforming the piezoelectric AC voltage into a DC voltage. Subsequently, the accumulated charge was harvested into an 8 nF mica capacitor. Following the charge of the capacitor, the circuit underwent a brief transformation, redirecting its energy either towards an oscilloscope or illuminating a sequential series of seven LED diodes via a short circuit.

## 3. Results

**Figure 7:**
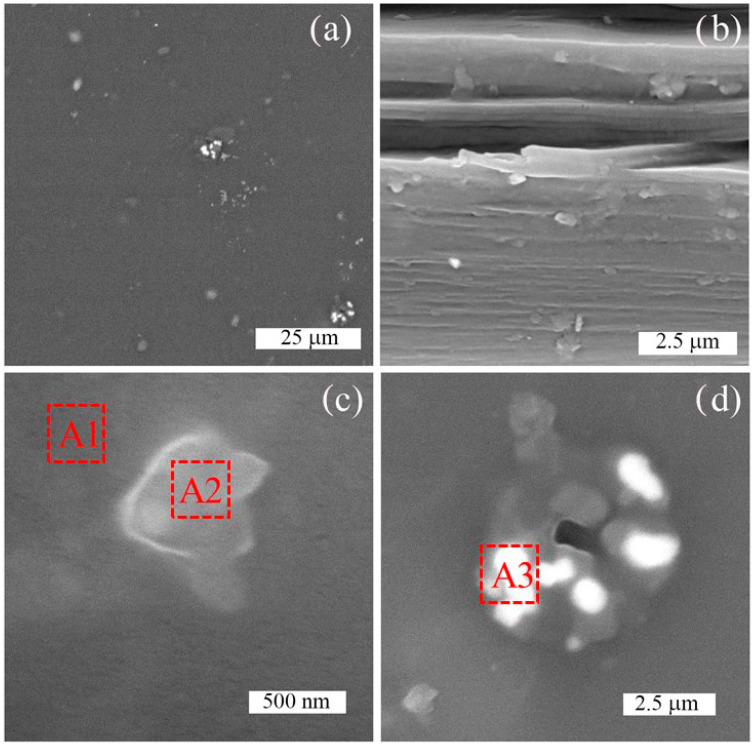
SEM analysis of (**a**) surface, (**b**) cross-section, and (**c**,**d**) detailed views of two particulate fillers identifiable in the polymer matrix. The red numbered squares show the places where the EDAX analysis was performed.

The SEM analysis revealed that the particles of the processing aid were distributed in the BOPET film, while EDAX analysis was used to determine their chemical composition. The filler particles on the surface of the BOPET film were observed as light spots (Figure 7a). The different intensities of the emitted light indicate the different chemical natures of the dispersed particles. They have a broad distribution of diameters, ranging from units of micrometers to approximately 500 nm. Particular solid particles were also observed in its cross-section (Figure 7b), which means that they were distributed in the bulk, and thus were admixed into the polyester melt.

The details of the two typical types of particles found in the BOPET film are presented in the lower parts of Figure 7c,d. The first represents one solid particle and the other is an agglomerate formed by the clustering of smaller particles.

The results of the EDAX analysis are depicted in Table 1; in area A1, only C and O elements were found, corresponding to the pure polymer matrix, and area A2 contained silicon as evidence of SiO_2_. There were also traces of other elements, such as F, Na, Cl, and K. A3 areas showed significant representation of elements, such as F, Na, Cl, and K, and traces of Si and Ca. These can represent other process ingredients, such as antiblockers and sliding additives such as waxes, as well as surfactants (CaCO_3_), for better sliding properties. Therefore, from the perspective of piezoelectric properties, BOPET can be considered as a polymer composite containing piezoelectric SiO_2_ particles distributed in a polyester polymer matrix.

**Figure 8:**
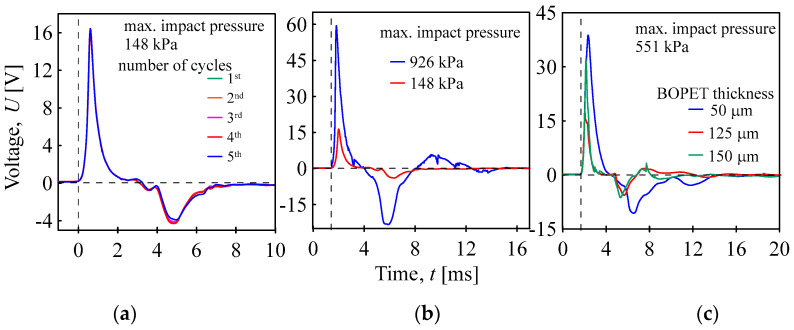
Piezoelectric responses of the BOPET film to pressure pulses after pendulum impact; five identical cycles (**a**), two different maximum pendulum impact pressures (**b**), and thickness of BOPET film (**c**).

The electrical reaction of BOPET to mechanical compressive stress was produced by employing a pendulum to create mechanical vibrations. In a single impact of the pendulum, the subject sample experienced compression, leading to an initial voltage of approximately 16 V (the maximum measured pressure during the impact was ~148 kPa, and the impact velocity was ~2.1 m/s). As the pendulum recoils, the BOPET film relaxes, producing a voltage of the opposite polarity. The subsequent diminishing elastic vibrations of the film also resulted in voltage oscillations, which dissipated within 7 ms, as shown in Figure 8a. Furthermore, the effect of the amount of applied compression on the piezoelectric response was investigated. Figure 8b shows two (148 kPa and 926 kPa) of the seven tested pressures. The effect of the thickness of the BOPET film for a maximum applied pressure of 551 kPa (Figure 8c) resulted in 38 V for the 50 µm film, 32 V for the 125 µm film, and 16 V for 150 the thick film.

**Figure 9:**
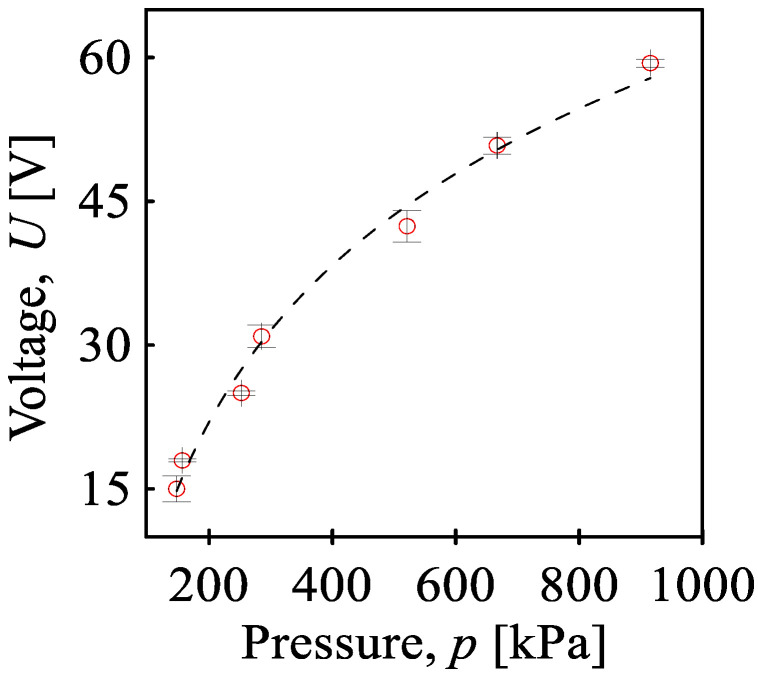
Generated maximum piezoelectric voltage on the maximum mechanical pressure caused by the pendulum impact on BOPET film.

The measurement confirms the assumption that by increasing the amount of compressive stress, the piezoelectric response increases from 16 V at 148 kPa to 60 V at 926 kPa. A greater level of deformation of the SiO_2_ crystals leads to a higher piezoelectric voltage. The dependence of the maximum piezoelectric voltage on the maximum mechanical pressure caused by pendulum impact is shown in Figure 9. The generated voltage does not correspond linearly to the applied pressure, but the effect of pressure gradually decreases from values around 0.1 V/kPa to 0.05 V/kPa. A typical polymer compressive stress curve (dependence of strain on the applied stress) is also nonlinear. As the pressure increases, the influence of the pressure on the deformation decreases, and the material resists the deformation more. The piezoelectric properties are determined by the deformation of the crystalline SiO_2_ lattice when the deformation of the polymer matrix is transferred. Thus, when the deformation of the polymer matrix is smaller, the effect of pressure on the piezoelectric response is also reduced.

**Figure 10:**
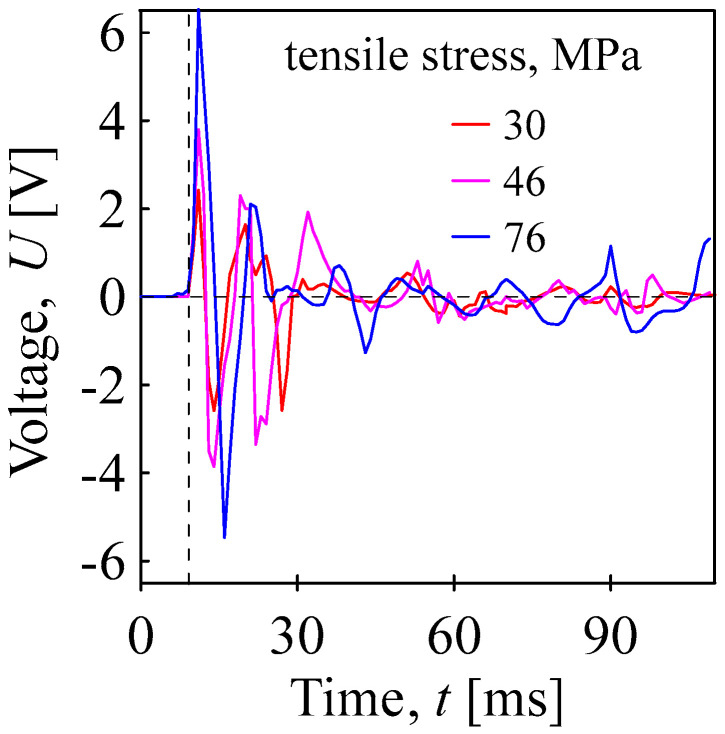
Piezoelectric responses of the BOPET film to tensile stress pulses for different stresses indicated.

The electrical reaction of BOPET to mechanical tensile stress pulls for different applied maximal stresses is presented in Figure 10. The specimen was elongated, followed by relaxation in length with time dependency as vibrations. The initial voltage of approximately 2.4 V for ~30.3 MPa and a velocity of ~2 m/s was measured.

**Figure 11:**
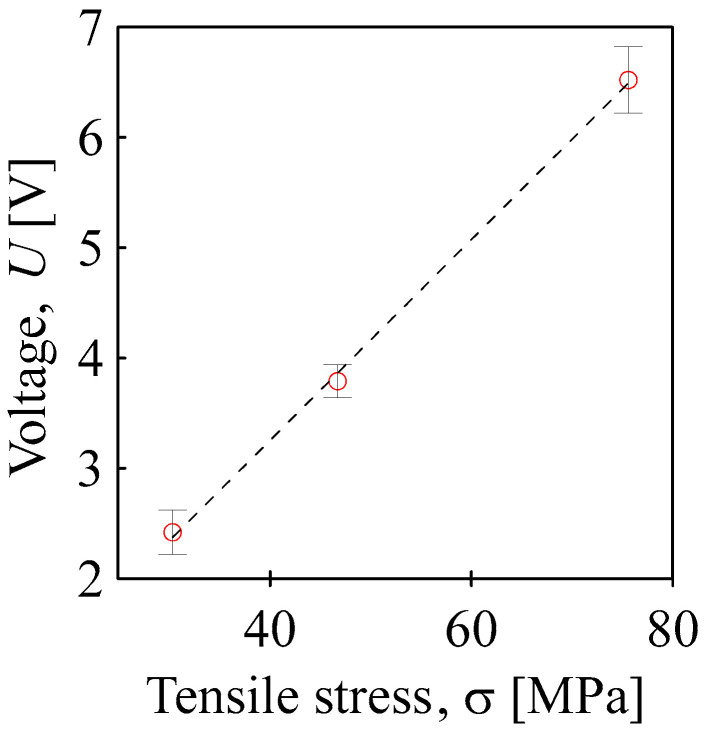
Maximum piezoelectric voltage generated on the tensile stress of BOPET sensor.

Similar to the case of compressive stress, the piezoelectric response increased with the magnitude of the applied stress and the applied tensile stress, as shown in Figure 11. However, the BOPET sensor prepared in this way was much less sensitive to applied stress than it was in the case of pressure stress. The measured tensile stress dependency was linear, with a slope of approximately 0.09 V/MPa.

**Figure 12:**
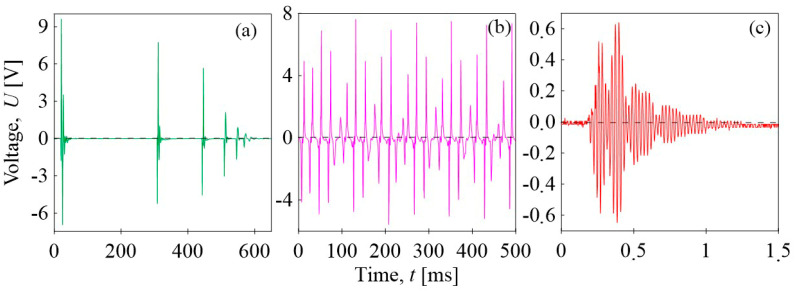
Electromechanical conversion by the BOPET film as a self-powered polymer sensor transforming mechanical stimuli into corresponding electrical signals: (**a**) free fall impact of a glass ball, (**b**) vibration by sieving machine, and (**c**) the vibrational pressure stress derived by the sonotrode.

The BOPET film can serve as a mechanical–electrical transducer, as illustrated in Figure 9, showing its application in pendulum impact detection. Additional examples of this self-powered mechanical stimuli sensor are shown in Figure 12. The BOPET sensor, assembled according to the scheme in Figure 5a, was subjected to mechanical stimuli such as vibrations by a sieve analyzer, free-fall impact of a glass ball, or vibrational pressure stress induced by the sonotrode. In all scenarios, BOPET efficiently converted mechanical stimuli into a corresponding electrical signal, demonstrating sensitivity, real-time responsiveness, reversibility, and reproducibility. The response to the free fall impact of a glass ball weighing 52 g at a speed of 4.1 m/s attained 9.6 V, as shown in Figure 12a. In the other responses, the subsequent impacts of the ball caused by elastic rebound from the sensor with subsequent attenuation during repeated impacts were recorded. Figure 12b shows the response to a mechanical pressure stimulus derived using a sieve analysis machine. The sequences of cyclically repeating vibrations were observed with an approximate sequence frequency of ~17 Hz. A highly sensitive response of the sensor was recorded both during its compression and subsequent relaxation within a range of maximum voltages of 8 V. Finally, the vibrational pressure stress from the ultrasound region derived by the sonotrode was measured. The measurement was performed in pulse mode; Figure 12c depicts one pulse with a converted electrical signal with a frequency of 22 kHz and a maximum amplitude of approximately. ±60 mV.

**Figure 13:**
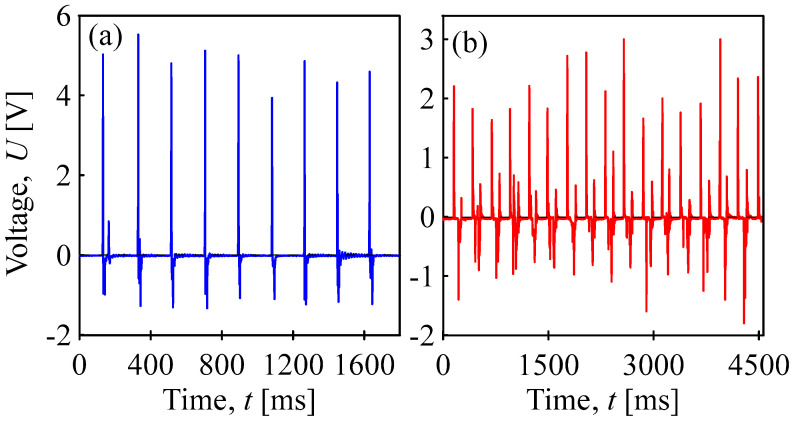
Electromechanical conversion by the BOPET film as a self-powered polymer sensor transforming mechanical stimuli into corresponding electrical signals applied in the area of wearable electronics: (**a**) tapping of finger, blue; (**b**) steps taken with the sensor placed under the heel, red.

Examples of self-powered mechanical stimulus sensors for wearable flexible electronics are shown in Figure 13. The response of the sensor to the tapping of the finger is sensitive and reversible, and the maximum generated electric voltage is approximately 5 V; when steps are taken with the sensor placed under the heel, it generates a voltage of more than 2 V.

**Figure 14:**
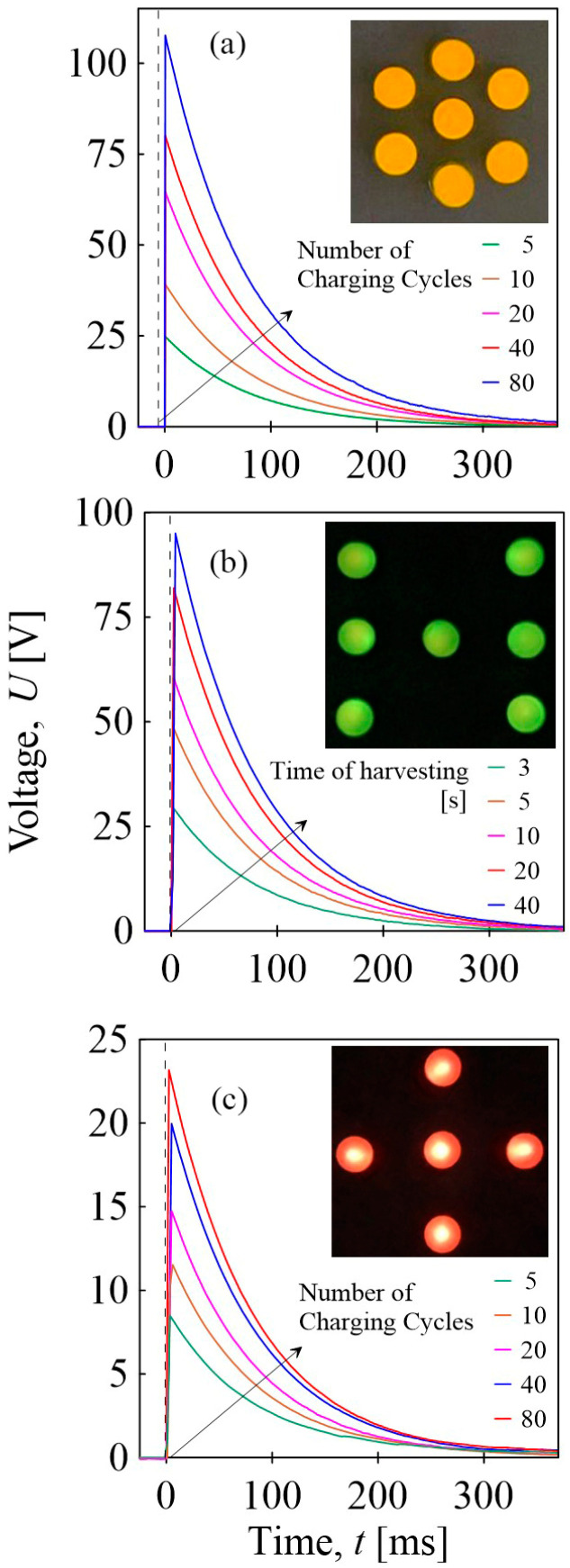
The short-circuited voltage of the charged capacitor by: (**a**) pressure pulses after pendulum impact onto BOPET foil, (**b**) vibrations delivered by sieve analysis apparatus, and (**c**) tensile stress pulses. Light flash of seven yellow LED diodes connected in series after 10 impact cycles, seven green ones after 10 s of vibration harvesting, and five red diodes after 80 tensile pulse cycles.

The pendulum impact on the BOPET film and vibrations delivered by sieve analysis was converted into an electric signal. This energy can be stored simultaneously, turning mechanical energy into electricity through mechanoelectrical conversion. The generated piezoelectric voltage is in the form of AC, and for effective utilization, it must be rectified into DC. This was achieved using the Graetz Bridge, a full-bridge rectifier. The final DC charge can then be stored in the capacitor. After charging the capacitor, it can be short-circuited. Figure 14 shows the time-dependent short-circuited voltage, which was stored in the capacitor for varying durations of the sieve analysis apparatus vibration detection/conversion, different numbers of pressure pulses after pendulum impact, and tensile stress pulses. After the short circuit, the voltage response reaches a significant maximum and then decays exponentially to zero over time. The discharge times for all harvesting times ranged up to 350 ms after a short circuit. The lowest values were obtained for tensile stress pulses (above 20 V after 80 cycles). The size of the peaks increased with the number of pendulum impacts, reaching approximately 107 V for 80 impact cycles, and the duration of the sieve analysis vibration was 95 V for 40 s. The demonstration of the potential to utilize stored energy is evident in the flashing of seven yellow LED diodes connected in series for impact cycles, seven green LED diodes for energy from vibrations, and five red LED diodes for tensile pulses with a lighting duration of approximately 10 ms.

## 4. Discussion

This article reports an important finding that polymer packaging materials can have unexpected properties, and their waste can be further employed in the area of sensors and energy harvesting.

The idea that polymeric packaging waste can be converted into electrical energy should be understood as a way to produce renewable energy. A renewable energy source is defined as energy that can renew itself on a human timescale. In connection with this, various types of waste have been considered, especially those leading to caloric utilization. This is how biomass, biogas, and waste incineration are used, or hydrogen is produced by the pyrolysis of waste plastics [31]. Solid municipal waste is also a source of renewable energy instead of being landfilled [32]. Alternatively, fuel for ignition engines is produced from waste plastics [33].

The intricate process of the production of biaxially oriented polyethylene terephthalate films, primarily intended for food packaging applications, unfolds through the biaxial stretching of amorphous polyethylene terephthalate and its subsequent crystallization under tension within the heat exposed. The heat-setting stage is crucial to prevent the film from reverting to its initial unstretched state and firmly anchoring the alignment of the polymer chains in the film direction. Notably, this molecular alignment triggers the initiation and growth of the embryonic crystals.

Examination of the structure revealed a consistent edge-on crystalline orientation between the rigid amorphous fraction and mobile amorphous fraction [34]. The presence of the rigid fraction in BOPET plays a crucial role in reducing the polymer chain mobility, consequently impacting the potential conductivity of the charge carriers. All the lamellar crystals in the BOPET films exhibited an edge-on configuration. This characteristic minimizes the hindrance of charge-carrier conduction imposed by these crystals [35].

Additives are incorporated into polyethylene terephthalate and other thermoplastics to modify their processing properties. However, in this study, it is demonstrated that they can have a different function in the recycling of polymer packaging.

In the case of the BOPET film, SiO_2_ was added to pure polyethylene terephthalate as an additive to reduce the generation of unwanted static electricity during film winding. Using BOPET waste, we obtained a polymer composite with a PET matrix, in which the piezoelectric filler was distributed. Therefore, the entire packaging polymer film was piezoelectric.

When the film is mechanically stimulated, mechanical energy is converted into electricity. This conversion is highly sensitive, and the response is reversible in real-time deformation. Moreover, it is a function of the applied mechanical stress, and the generated electrical voltage increases with increasing stress. In this way, the waste material is applicable as a self-powered sensor of mechanical stimuli, where the further generated signal can serve as effective electrical energy through the harvesting process.

As previously mentioned, poly (vinylidene fluoride) is a rare polymer material that serves as a piezoelectric energy harvester. The PVDF-based nanogenerators usually provide very good output values; a piezoelectric organic–inorganic hybrid PVDF-TrFE nanocomposite film with BaTiO_3_ nanoparticles showed output voltages of 59.5 V, but at a low compression pressure of 100 N [16], P(VDF-HFP) filled with polyaniline and (CH3NH3PbI3) has an excellent output power of 8.2 nW at a pressure amplitude of ∼14 kPa [10].

However, these materials are purpose-built, expensive, toxic, and chemically unstable. However, the material proposed in this paper, where approximately 60 V for a pendulum impact with a pressure of 926 kPa was obtained, solves some of the drawbacks associated with PVDF-based nanogenerators, as BOPET is a harmless material intended for contact with food.

Currently, flexible materials can effectively serve as self-powered sensors for signaling external stimuli. Due to their convenient elastic properties, they can be utilized in high-tech areas, such as soft robotics [36] and wearable electronics [37]. As a source of energy based on ever-improving methods of harvesting, innovative procedures can be applied to obtain higher-energy performance from low-energy sources, for example, by grouping and connecting them in series and blocks. Examples with a potential perspective in this area include piezoelectricity for the conversion of wind energy [38] or the conversion of mechanical energy of vehicles on highway power supply systems [39].

## 5. Conclusions

The aim of this study was to use a waste BOPET film to prepare a mechanical stimuli sensor and provide mechanoelectrical conversion for useful electrical energy generation. Due to its orientation and stress-induced crystallization of polymer chains, BOPET acquires very good mechanical properties, which are beneficial for the durability of sensors and the long-term generation of electrical energy from sources such as (otherwise wasted) vibrations of machines. Due to the flexibility of BOPET, such sensors are suitable for wearable electronics. The SiO_2_ particles added to BOPET as a technological additive during the production of films for the packaging of food as well as technical products, including electronics, bring piezoelectricity to BOPET. The aforementioned possibility of contact with food shows that it is a polymer composite, which is not toxic or harmful to human health. This is another great advantage of BOPET sensors if one considers that a number of solutions for piezoelectric polymer composites are based on harmful inorganic fillers. In addition to its excellent mechanical properties, BOPET shows good temperature stability, and according to DSC, the material shows no changes up to the melting temperature of the crystalline phase of approximately 245 °C; thus, it can be considered a high-temperature resistant material. Its chemical resistance should also be mentioned, as it dissolves only in strong solvents, such as trifluoroethanol or fluoroacetic acid. Finally, it is stable when exposed to oxygen, ultraviolet light, and moisture.

## Figures and Tables

**Table 1:** Results of EDAX analysis of particulate fillers found in the tested polymer waste packaging analyzed in areas A1–A3 depicted in Figure 7.

Area in SEM	Element	Weight %	Atomic %
A1	C	90.76	92.9
	O	9.24	7.1
A2	C	82.88	89.08
	O	8.36	6.74
	Si	5.56	2.6
	traces F, Na, Cl, K	-	-
A3	C	80.52	90.5
	O	3.19	2.69
	F	1.41	1
	Na	1.36	0.8
	Cl	5.67	2.16
	K	6.65	2.29
	Traces Si, Ca	-	-

## Data Availability

Data obtained throughout the study were stored according to the TBU Data Management Plan and are available upon request from the corresponding author.
